# Nutrient Adequacy of Global Food Production

**DOI:** 10.3389/fnut.2021.739755

**Published:** 2021-11-29

**Authors:** Canxi Chen, Abhishek Chaudhary, Alexander Mathys

**Affiliations:** ^1^Laboratory of Sustainable Food Processing, Department of Health Science and Technology, Institute of Food, Nutrition and Health, ETH Zurich, Zurich, Switzerland; ^2^Department of Civil Engineering, Indian Institute of Technology (IIT) Kanpur, Kanpur, India

**Keywords:** nutrition, food production and consumption, food security, global food systems, sustainable development goals

## Abstract

A major challenge for countries around the world is to provide a nutritionally adequate diet to their population with limited available resources. A comprehensive analysis that reflects the adequacy of domestic food production for meeting national nutritional needs in different countries is lacking. Here we combined national crop, livestock, aquaculture, and fishery production statistics for 191 countries obtained from UN FAO with food composition databases from USDA and accounted for food loss and waste occurring at various stages to calculate the amounts of calories and 24 essential nutrients destined for human consumption. We then compared the domestic production quantities of all nutrients with their population-level requirements estimated from age- and sex-specific intake recommendations of WHO to assess the nutrient adequacy of the national food production. Our results show inadequate production of seven out of 24 nutrients (choline, calcium, polyunsaturated fatty acids, vitamin A, vitamin E, folate, and iron) in most countries, despite the overall adequacy of the total global production. High-income countries produce adequate amounts of dietary nutrients in general, while the foods produced in low-income countries mainly comprising roots and cereal products often lack in important micronutrients such as choline, calcium, and vitamin B12. South Asian food production barely fulfills half of the required vitamin A. Our study identifies target nutrients for each country whose domestic production should be encouraged for improving nutritional adequacy through interventions such as increasing the production of foods or fortified foods that are rich in these inadequate nutrients while not undermining the local environment. This assessment can serve as an evidence base for nutrition-sensitive policies facilitating the achievement of the Sustainable Development Goals of zero hunger and good health and well-being.

## Introduction

It is the basic need of people around the world to have enough foods to support their healthy and active lives. However, the widely prevalent food insecurity and malnutrition in all forms around the world acts as a barrier in achieving the Sustainable Development Goal (SDG) 2 Zero Hunger and 3 Good Health and Well-being (1–3). Evidence-based interventions across different components in the global food systems including production, distribution, processing, and marketing can help improve nutritional outcomes and achieve SDGs (4–6). Above all, sufficient food production is fundamental in supporting adequate consumption of nutrients by the population (7). Food production systems nationally and globally must therefore be aligned with the nutritional needs of the people.

Assessing the balance between food production and nutritional requirement could also facilitate resource use efficiency and environmental sustainability. The production of food items that contribute little to nutrition can be reduced and instead the production of nutrient dense items can be incentivized through government policies which will improve the nutritional security without extra cost to the environment (8). Global food systems have been shown to be responsible for significant environmental footprints in terms of greenhouse gas emission, water and land use, fertilizer application and associated pollution (8–12). Food production is the biggest driver of natural habitat loss and the associated species extinctions, with about 50% of the world's habitable land area already used for agriculture (13) and food system transformation was placed at the center for reversing the decline in biodiversity (14, 15). However, recent increases in per capita income and agriculture yields is driving skewed increase in the demand for high-impact animal-sourced food products, consequently exacerbating the environmental damage (10).

Meanwhile, the prevalence of inadequate nutrient intake and the consequent diet-related non-communicable diseases and obesity shows that the current food systems are not fully addressing nutritional and health requirements of the people around the world (16, 17).

Previous studies have investigated the nutrient quantities available in the national food supply and matched them with the nutritional requirements to inform food policies that can tackle the food insecurity and malnutrition concerns regarding the current diets. Most such studies have focused on just calories (18–20) or a couple of nutrients (21–23) while others have investigated the sufficient production of a limited number of macro and micronutrients (16, 24, 25). Only a few studies have considered the comprehensive nutritional factors involved (26–28). Nutrients such as choline and polyunsaturated fatty acids (PUFAs) are widely acknowledged for their crucial roles in human health (29–32) but they rarely feature in the context of food and nutrition security.

Based on the data from the United Nations' Food and Agriculture Organization (FAO) country-specific Food Balance Sheets (FBS), many studies have estimated the available nutrient quantity per country (16, 18, 24–28, 33) but were limited in their resolution of food commodities. Compared to the FAO FBS that only has around 98 food items, the FAO production statistics at the national level has more disaggregated and high-resolution data with over 170 individual food items and hence can provide more accurate estimates of nutrient availability at the national level (34).

The surplus or deficit in a region's food production is automatically coupled with downstream patterns like distribution, trade, pricing, and consumption. There has been an increasing dependence on international trade for national food security (35), but potential and ongoing shocks (e.g., natural disasters, pandemics) present considerable risks to the resilience of the global food systems and nutrient security. It is thus important to explicitly attribute the dietary nutrient sources to different supply components (domestic production, net trade, and stock variation) for assessing the risks.

Unlike previous studies using food balance sheet (FBS) data, Geyik et al. (34) used highly disaggregate crop, livestock, and seafood production data from FAO (36) to compare the total nutrients obtained from primary food production with the respective nutritional requirements of the national population to calculate the adequacy of domestically produced calories and six nutrients (protein, iron, zinc, vitamin A, vitamin B12 and folate). After correcting for food used as animal feed and applying conversion factors for food items that need primary processing before being edible (such as palm oil and rice), they found that the domestic production is insufficient to meet the nutritional requirements in up to 120 countries especially for vitamin A. However, their study was limited to six nutrients and they did not correct the raw production amounts for food loss and waste occurring in different stages and non-food utilization (e.g., seed, biofuels, etc.) losses and thereby overestimating the food available for human consumption.

Building on the aforementioned study, here we account for above loss factors to better reflect the net production quantities that finally contribute to human nutrition in each country and also carry out a more comprehensive assessment by considering 25 dietary nutrients instead of six. We calculate the nutrient adequacy of domestic food production for 191 countries by combining the disaggregated food production data from FAO with the high-resolution food composition tables from United States Department of Agriculture's (USDA) standard reference database (37) and GENuS model (26), and country-specific nutrient requirements estimated from age- and sex-differentiated recommendations from the WHO and UN population statistics.

## Methods

Based on the food production data from FAO (36), we calculated the ratio of the produced and required nutrient amounts for each country and defined it as adequacy ratio to identify the potential gaps or surplus in dietary energy and 24 essential nutrients for 191 countries. For a given nutrient, an adequacy ratio value lower than one indicates that the nutrient amount provided by the domestic food production is less than the level required for the population of that country.

### National Nutrient Production

We acquired the national production data of 119 crop and 27 livestock items reported in FAO Statistics Division (36) and of 28 seafood items including those from the capture fisheries and aquaculture (in tons) from the FAO Fisheries and Aquaculture Department for the years 2011–2014 (38).

The nutrients from raw food production do not all contribute to human food consumption. Some are converted during primary processing (e.g., from palm fruits to palm oil), utilized for animal feed, seed, other non-food products such as oil for soap, or lost along the supply chain. Hence, we adjusted the primary FAO production quantities by considering the changes or losses to better estimate the actual nutrient production going toward final human consumption.

Starting with the raw production quantities, the amount of animal feed from national food production can be calculated by multiplying the production amount per food item per country by the percentage of produced foods used for animal feed (i.e., feed-to-production ratio). Details on the methodology used to estimate the food to feed ratio per food item per country can be found in Geyik et al. (34). Similarly, we applied correction factors for seed use, other non-food use (e.g., oil for making soap), and loss at the storage and transportation stages. We also estimated the amount wasted at the consumption stage using the region- and food-group-specific wastage portions (39). For food items that require primary processing to align with the items in the nutrient composition dataset, we applied the conversion factors (40) at the commodity level to estimate the output amount after processing following Geyik et al. (34). Lastly, the inedible parts of the food products (e.g., shells of nuts) were excluded from the production quantity by using the refuse factor in the food composition tables (36).

Subtracting all the above losses and wastes from the total production amount provided us with the net production amount (i.e., amount destined for human consumption) of each food item *f* in each country *c* in the year *t* (Equation 1):


(1) $$\begin{array}{l} Net\;production_{f\text{​},c,t}=(total\;production_{f\text{​},c,t}\;-feed_{f\text{​},c,t}-seed_{f\text{​},c,t}-\\ \;other\;use_{f\text{​},c,t}-loss_{f\text{​},c,t}-waste_{f\text{​},c,t})\\ \;\times \;conversion_{f}\times \left(1-refuse_{f}\right) \end{array}$$

The food items from the FAO databases were then matched with the items from the USDA food composition database (36) to estimate the production amounts per country per year for calories and 24 essential nutrients. When USDA did not have the matched food item or nutrient density values (e.g., for choline, vitamin K), the food composition tables of GENuS model (26) and data from USDA FoodData Central (41) were used. For commodities (e.g., wheat) with various subspecies (e.g., hard wheat, soft wheat, red wheat, white wheat, spring wheat, winter wheat, durum wheat) in the food composition tables, we took the average of the nutrient values of the subspecies. Our matching followed the rules of previous papers (24, 26, 34). For each of the individual food item, we thus obtained the nutrient density, i.e., amounts of calories and 24 nutrients (e.g., kcal for energy, mg for iron) per ton edible part. Multiplying the nutrient density with the net production amount (i.e., total production minus the loss) of a particular food item provided us with the net nutrient production from that food item.

The above procedure for estimating the amount of nutrient *k* produced from food item *f* in country *c* in the year *t* is expressed in Equation 2:


(2) $$\begin{array}{l} {Net\;nutrient\;production}_{k,f,c,t}=\left[{Net\;production}_{f,c,t}\right]\times \\ \;{Nutrient\;density}_{k,f} \end{array}$$

### National Nutrient Requirements

We calculated the population-weighted required intakes of 24 nutrients and energy for 191 countries in the years 2011–2014 by combining the age- and sex-specific daily nutrient requirements and the age- and sex- subgroup data from the Population Division of United Nations (42). We adopted the Recommended Nutrient Intakes (RNI) that represents the daily nutrient intake amount for meeting the requirements of 97.5% of healthy individuals in a particular population group and is tailored to global assessments. With the variability in the intake recommendations for different sex and age groups, a country's demographic structure (i.e., the population size of each sex and age group) determines our estimates of the national nutritional needs for all populations.

For 17 of the total 25 nutritional factors, we adopted the age- and sex-specific RNIs from WHO guidelines (43–45). The recommendations from the Institute of Medicine (32, 46–48) were applied for phosphorus, manganese, copper, choline, potassium, and fiber, because the WHO guidelines do not include them. Following other studies (25), we used Adequate Intake (AI) which is the level of intake assumed to be adequate for healthy individuals when an RNI is not available for certain nutrients or population groups. In line with previous assessments (8, 16, 27), we considered the proportion of calcium sourced from drinking water and adopted an average water intake of 1.7 L per capita per day with a calcium concentration of 42 mg/L in our analysis. The RNI of PUFAs were derived from the lower limit of the 6–10% recommended PUFAs contribution to total daily energy intake (29) and the 9 kcal/g energy density of fat.

We also considered the special needs of pregnant women and acquired the estimates for the annual nutrient requirement of pregnant women by using the country-specific five-year births by age group of mothers provided by United Nations (42). We assumed an average gestation period of 280 days when estimating the population-level nutrient requirement per year (i.e., a normal RNI for the remaining 85 days).

Nutritional guidelines provide a range of recommendations for iron and zinc owing to the variance in the bioavailability among individuals. In line with previous practices (24, 27), we assumed a medium level of bioavailability in our estimations. Similarly, energy requirements are associated with the activity level of individuals, and we assumed a moderate activity level in our analysis.

Following Geyik et al. (34), we estimated the requirements for non-adult subgroups by using the energy and protein requirements in different age groups under global reference weights. To estimate the protein requirements for adults (aged >18 years) dependent on the body mass (44, 45), we matched the recommended amount per unit of human body weight with the national average adult body weight calculated using data reported by the NCD Risk Factor Collaboration (49, 50). This anthropometric information was also applied to determine the per capita average energy requirement of each country by sex and age specific recommendations for a moderate physical activity lifestyle (45).

### Assessing the Nutritional Adequacy of Food Production

Three indicators were used to assess the nutritional quality of a country's food production. To figure out how domestic food production fulfills the nutrient needs of a national population, we calculated the adequacy ratio (34) using the adjusted national production of various food items (*f*) and population-weighted per capita requirement per country for each of the 24 nutrients and energy. The adequacy ratio for the nutrient *k* in country *c* for the year *t* was calculated through Equation 3:


(3) $$\begin{array}{l} {Nutrient\;adequacy\;ratio}_{k,c,t}\\ =\;\frac{\sum_{f}{Net\;nutrient\;production}_{k,f,c,t}}{{average\;nutrient\;requirement\;per\;capita}_{k,c,t}\times {population}_{c,t}} \end{array}$$

A ratio value above one indicates that the domestic production is enough or even surplus to meet the nutrient requirements of national population, and values <1 indicate that the nutrients embedded in national food production are less than the national requirement.

As our second indicator, we defined the minimum value of all 25 nutrient-specific adequacy ratios as the Food Production Adequacy per country *c* (Equation 4):


(4) $$\begin{array}{l} {Food\;Production\;Adequacy}_{c}\;=\;\min\;\left(Nutrient\;adequacy\;{ratio}_{k,c}\right) \end{array}$$

For the third indicator Population Share with Adequate Nutrients (PAN), we followed the population-weighted estimated average requirement (EAR) “cut-point” approach assuming a lognormal distribution of per capita available nutrient levels within a country constructed by the mean per capita produced amount and the coefficient of variation (CV) of each nutrient (28). Out of the 24 nutrients, we applied the CVs that have been used in previous studies for 16 nutrients ranging from 0.25 to 0.45 (28, 51, 52) and assumed a CV of 0.25 for those with no individual CV reported. In terms of dietary energy, we adopted the latest country-specific CVs (53). For countries with missing CVs, we adopted the mean of available CV of other countries in the same region. The population-weighted EAR was established based on age and gender specific nutritional recommendations and the population size of each subgroup for each country. The average PAN score of the 25 nutritional factors was regarded as the overall PAN for each country.

## Results

### Dietary Nutrients in Global Food Production

According to the production statistics of 174 crops, livestock, and aquaculture food commodities and the losses and waste from the food systems, the global food production per year supplied around 8,000,000 billion kilocalories for human consumption (Table 1). This dietary energy quantity is estimated to be as much as 6% of the world energy supply (13,304 million tons of oil equivalent) in the same period according to the statistics from the International Energy Agency (54).

**Table 1:** Global dietary nutrient production and adequacy.

**Energy and Nutrients**	**Production**	Production surplus^*^	**Adequacy ratio**		**% of all countries with adequacy ratio <1**	**% of global population living in countries with adequacy level <1**
	**(kcal or ton per year)**		**Global**	**Median of national values**		
Energy	8.0E+15	1.9E+15	1.31	1.02	48	16
Protein	2.5E+08	1.3E+08	2.03	1.52	28	6
Zinc	4.5E+04	2.9E+04	2.84	1.90	21	2
Iron	5.8E+04	1.4E+04	1.32	0.94	54	23
Calcium	2.3E+06	−1.2E+05	0.95	0.62	70	76
Selenium	5.6E+02	4.8E+02	7.69	4.00	7	1
Magnesium	1.8E+06	1.3E+06	3.38	2.55	17	3
Vitamin C	3.5E+05	2.4E+05	3.17	2.23	19	4
Thiamine	7.1E+03	4.3E+03	2.57	1.68	25	5
Riboflavin	4.8E+03	1.9E+03	1.67	1.26	39	19
Niacin	7.5E+04	3.9E+04	2.06	1.58	27	5
Vitamin B6	8.5E+03	5.3E+03	2.64	2.06	16	2
Pantothenate	2.0E+04	8.1E+03	1.67	1.25	35	9
Vitamin B12	1.1E+01	5.5E+00	1.97	1.54	32	33
Folate	1.2E+03	2.7E+02	1.28	0.80	68	37
Vitamin A	1.4E+03	−3.1E+01	0.98	0.70	66	63
Vitamin E	3.5E+04	1.4E+04	1.67	0.85	59	48
Vitamin K	6.7E+02	5.5E+02	5.39	2.27	15	3
PUFAs	4.4E+07	2.8E+06	1.07	0.57	70	72
Phosphorus	5.5E+06	3.5E+06	2.74	1.84	21	4
Manganese	3.3E+04	2.8E+04	6.74	3.95	16	1
Copper	7.3E+03	5.2E+03	3.47	2.62	16	3
Choline	9.8E+05	−1.7E+05	0.85	0.63	79	62
Potassium	1.1E+07	3.1E+06	1.38	1.16	38	15
Fiber	1.3E+08	5.7E+07	1.76	1.31	37	11

*Adequacy ratio below 1 (for calcium, vitamin A, choline) suggests that the total global production is not enough to meet the world's population requirements*.

*See Supplementary Table S3 for the adequacy values per nutrient per country*.

* *A negative number indicates a production deficit for a given nutrient*.

*PUFAs, polyunsaturated fatty acids*.

Nutrient production patterns vary across different regions. Cereal products contribute to the largest portion of calories (45%) produced globally, followed by animal-sourced products and oil crops. Essential nutrients for human health are sourced from different food products depending upon the country. Supplementary Table S1, S2 show the contribution of 16 broad food groups toward total domestic production of each nutrient per country and region, respectively.

Cereals are responsible for 40% of protein, 60% of dietary fiber, and 34% of the vitamins and minerals produced globally. Vegetables, meat, dairy, and eggs are also the major suppliers of global dietary nutrients for human consumption. Globally, Vitamin A is mainly supplied by vegetables, dairy and roots but this varies depending on the region. For low-income countries or Sub-Saharan African region, roots are the top supplier contributing to two-thirds of the total vitamin A production. In contrast, root-sourced vitamin A is negligible in Europe & Central Asia where more than 80% of the vitamin A production is through dairy and vegetables. Fish production in East Asia & Pacific (excluding China) contributes to 14% of regional vitamin A outputs, whereas its contribution in the rest of the world is only around 3%.

Among the world's nutrient production, dairy products are responsible for one-third of the total calcium with the remaining sourced from a wide range of food categories, such as meat and fish (17%), soybeans (11%), vegetables (10%), and cereals (9%). Half of the dietary choline is supplied by animal products (49%) especially eggs (16%), and one-fourth is derived from cereals. For polyunsaturated fatty acids (PUFA), oil, cereals and animal-sourced foods are the key sources contributing by 43, 22, and 18%, respectively. In total, 99% of dietary vitamin B12 is disproportionally sourced from global animal products. Supplementary Table S1 shows the contribution of different food groups toward the total quantity of each dietary nutrient.

### Production Adequacy of Individual Nutrients

By comparing the domestic nutrient production quantities and the population requirements, we found that the total production of dietary calories and nutrients from global food systems are more than the total required amount for the world population, except for choline, calcium, and vitamin A (Table 1).

Global food systems supplied 31% more calories than the human dietary energy requirement over 2011–2014, but not all countries produced adequate energy from domestic food systems. Our analysis showed that 91 countries representing 16% of the world population had their calorie production below the population intake requirements. Supplementary Table S3 shows the adequacy ratio per nutrient per country. Selenium, manganese, vitamin K, copper, magnesium, vitamin C were estimated to be remarkably adequate in global food production with the amounts available for human consumption three to seven times that of the required estimates. Choline, calcium, and vitamin A showed the lowest adequacy ratios of 0.85, 0.95, and 0.98, respectively, followed by PUFAs (1.07) and folate (1.28). Protein was found to have global adequacy ratio of 2.03, and only 28 out of 191 countries showed an adequacy ratio below 1 (Supplementary Table S3).

**Figure 1:**
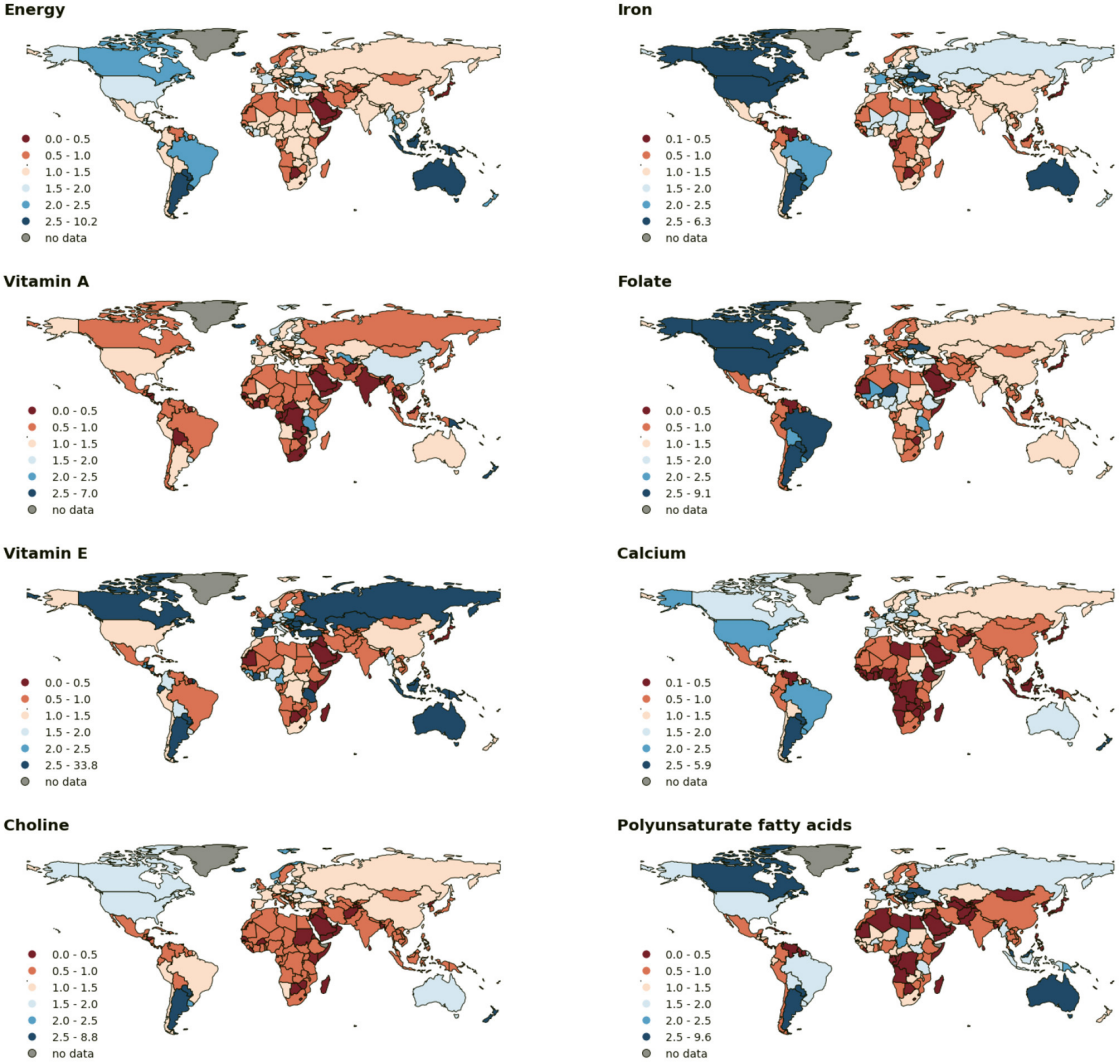
Country-specific Nutrient Production Adequacy ratios for eight selected nutrients. The numbers show the average production adequacy across 2011–2014. For a given nutrient, countries in darker red colors present lower adequacy in food production as compared to their national population requirement. Map breaks at a fixed interval in the range of 0.5–2.5 and extend to the minimum and maximum values out of this range for each nutrient.

Table 2 shows the region-specific production adequacy of the selected nutrients in our analysis. These eight nutrients are likely to have high gaps between the current production and the requirements according to our adequacy assessment at the global level (Table 1). Countries in Africa and Middle East were the hotspots of calorie production inadequacy with a regional adequacy ratio of 0.68 and also suffer from inadequate production of many essential macro- and micronutrients (Table 2, Figure 1). High-income countries had adequate food production on average to fulfill their requirements for all nutrients. For low-income countries, we found that calcium (regional average adequacy = 0.52), choline (0.59), and vitamin B12 (0.71), PUFAs (0.84), and vitamin A (0.93) from regional food production were inadequate. Moreover, the produced quantities of vitamin E (1.02), energy (1.05), and iron (1.08) barely reached the corresponding required levels as per the intake recommendations (Table 2). For lower-middle-income countries, we identified inadequate production of dietary calcium, folate, vitamin A, and choline. The production of vitamin A and choline was also below the required amount in upper-middle-income countries (Table 2).

**Table 2:** Regional nutrient production adequacy ratios for the selected nutrients.

**Regions**	**Energy**	**Iron**	**Calcium**	**Folate**	**Vitamin A**	**Vitamin E**	**PUFAs**	**Choline**
High income	1.29	1.73	1.47	1.35	1.09	1.63	1.31	1.17
Low income	1.05	1.08	0.52	1.36	0.93	1.02	0.84	0.59
Lower middle income	1.42	1.01	0.60	0.97	0.66	2.08	1.00	0.63
Upper middle income	1.67	1.59	1.24	1.71	0.79	2.80	1.48	1.05
East Asia & Pacific	1.95	0.87	0.57	0.70	0.68	3.28	1.33	0.68
Europe & Central Asia	1.34	1.77	1.38	1.19	1.19	3.19	1.47	1.13
Latin America & Caribbean	1.65	1.78	1.63	2.35	0.78	1.55	1.49	1.20
Middle East & North Africa	0.68	0.86	0.55	0.70	0.66	0.74	0.38	0.57
North America	1.75	2.75	2.05	2.76	1.10	1.77	2.07	1.65
South Asia	1.06	0.88	0.65	0.63	0.43	0.67	0.41	0.50
Sub-Saharan Africa	1.13	1.07	0.51	1.40	0.85	1.20	0.94	0.62
China	1.09	1.38	0.96	1.26	1.86	1.35	0.98	1.03
India	1.17	1.09	0.75	1.15	0.49	0.82	0.65	0.54

*Values above 1 indicate that the dietary nutrients in the regional food production meet the amount required by the people living in this region, whereas values <1 denote a gap between the regional production and requirement*.

*PUFAs, polyunsaturated fatty acids*.

Across different geographical regions, we found that Middle East & North Africa had a remarkable gap between their dietary nutrient production and population requirements. Food production there failed to meet the population requirements of calories and seven essential nutrients by 14–62%. South Asian food production is inadequate for ten dietary nutrients, of which PUFA and vitamin A showed severely low adequacy ratios of 0.41 and 0.43, respectively. The local production gaps in Sub-Saharan Africa were predominant with respect to calcium, choline, vitamin A, vitamin B12, and PUFAs. In contrast, North America and Europe & Central Asia were the self-sufficient regions in terms of energy and all 24 nutrients considered in this study, and Latin America & Caribbean showed adequacy in dietary nutrient production except vitamin A (0.78). Even for nutrients that are globally under-supplied such as choline and calcium, North America produced 65% and 105% more than the domestic nutritional needs, respectively (Table 2). Figure 1 shows the large variability in adequacy ratios across the 191 countries.

The food production in India could not fulfill the population requirements of the vitamin A (Nutrient Production Adequacy ratio = 0.49), choline (0.54), PUFAs (0.65), vitamin B12 (0.74), calcium (0.75), and vitamin E (0.82). Domestic food production in China was estimated to meet the national nutritional requirements for all nutrients considered in this study, except minor deficit in calcium (0.96) and PUFAs (0.98). Particularly, Chinese foods provided large amounts of vitamin A with an adequacy ratio higher than that of any other region in the world.

While many countries have surplus nutrient production (blue shade in Figure 1), domestic food production in countries such as Singapore, United Arab Emirates, Qatar, Bahrain, Puerto Rico is too less to fulfill any nutritional requirements on a population level, with adequacy ratios below one for all 25 nutrients in our analysis (Figure 1, Supplementary Table S3).

Among different countries, the United States showed the largest calcium and choline production surplus, accounting for around 123,000 tons and 33,000 tons per year, respectively. The calcium surplus was also high in Brazilian foods by 98,000 tons per year.

Iron which is associated with high deficiency prevalence among the global populations, was abundantly produced in United States, China, Brazil, and Argentina with a surplus in annual food production of 3,196, 3,355, 1,664, and 1,372 tons, respectively. Regarding vitamin A, China showed the largest surplus of 239 tons after meeting the domestic requirements. Vitamin A produced in populous countries such as India, Bangladesh, Indonesia, and Pakistan were far below the levels required for the people living there, with gaps ranging from 14 to 130 tons per year.

**Figure 2:**
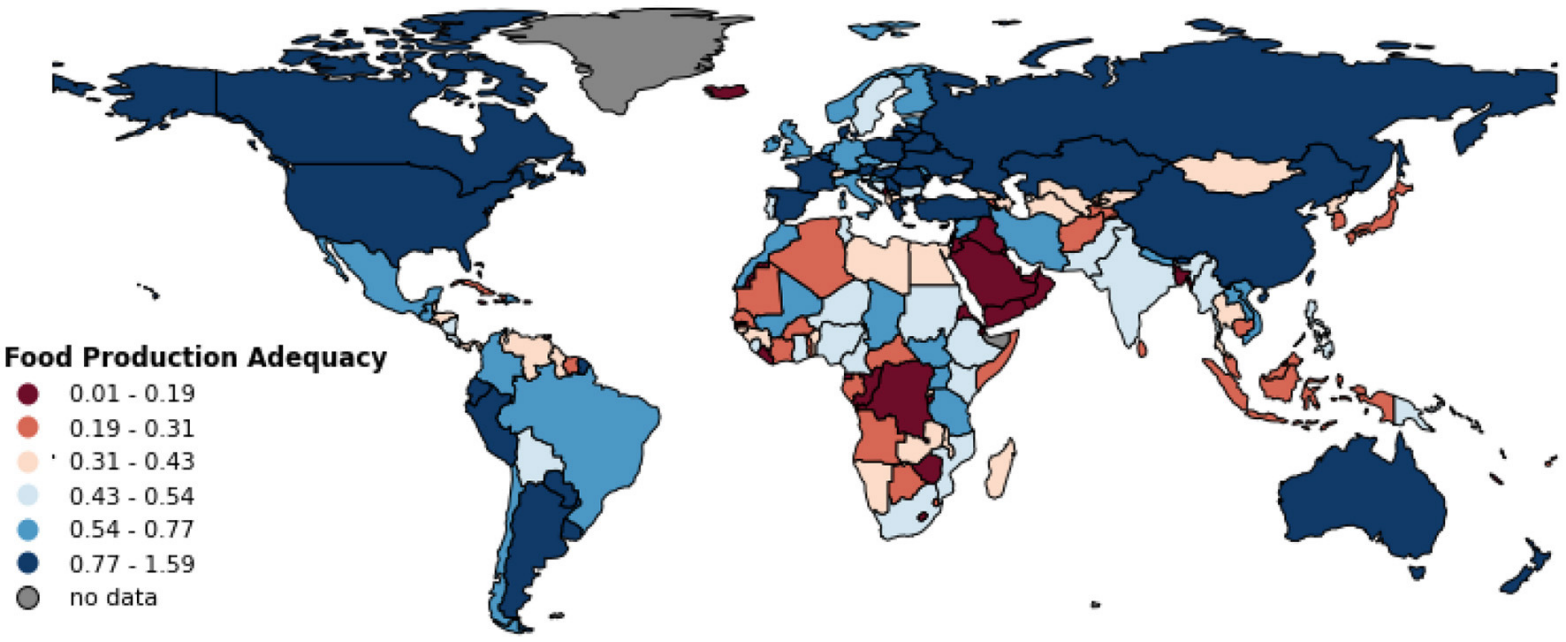
Country-specific Food Production Adequacy for the year 2011–2014. The values represent the minimum of the adequacy ratios for energy and 24 essential dietary nutrients per country. Symbology is based on quantiles classification method.

Regarding our second indicator (Food Production Adequacy) which is the minimum value of adequacy ratio across the 25 nutritional factors for a given country, we found that 179 out of 191 countries had values below one indicating that their present-day food production (year 2011–2014) was inadequate to meet the national nutritional requirements for at least one of the 25 nutritional factors considered here (Supplementary Table S3). The global median Food Production Adequacy score for the year 2011–2014 was 0.40. Figure 2 shows the countries classified into six groups according to their Food Production Adequacy values, and Supplementary Table S3 in supplementary information shows the score per country.

Countries such as Uruguay, Argentina, Australia, France, Ukraine, Lithuania, and New Zealand scored in the top quantile and marked in dark blue in Figure 2. Countries in Middle East, Africa, Southeast Asia, and Caribbean regions showed relatively low Food Production Adequacy values. PUFAs, vitamin A, calcium, and folate were the most frequent limiting nutrients in determining the national Food Production Adequacy. This means that the adequacy ratio for these nutrients were often very small, and thus associated with the low production adequacy ratios for many countries.

**Figure 3:**
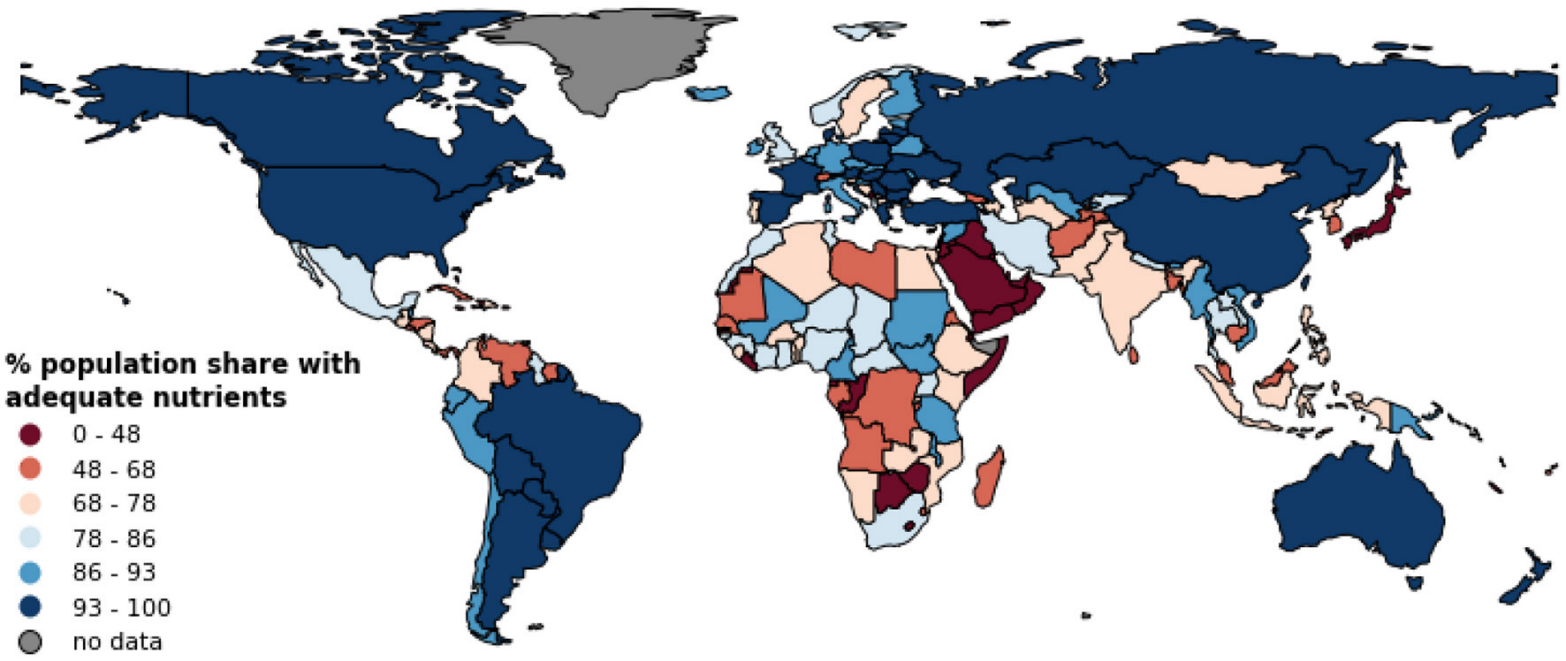
Population share with adequate nutrients (PAN) from the national food production. Symbology is based on quantiles classification method. See Supplementary Table S4 for PAN scores per country per nutrient.

For our third indicator Population Share with Adequate Nutrient (PAN), the world average score is 67%. As shown in Figure 3, the geographic pattern for the PAN indicator is similar to that of Food Production Adequacy with low scores for many African, Asian, and Caribbean countries.

## Discussion

By comparing the country-specific nutritional requirement estimates with the nutrients from domestic food production, this study shows that 94% of countries do not fulfill the population needs in terms of one or more of the 25 nutritional factors (i.e., Food Production Adequacy <1, Figure 2). Despite adequate calorie production in most countries, the provision of essential dietary nutrients such as choline, calcium, and vitamin A is often inadequate in most countries' domestic food production systems while the production amounts of polyunsaturated fatty acids (PUFA), folate, and iron barely meeting their respective total requirement (Table 1). This study also reveals the heterogeneity in the way countries produce different nutrients from different food sources (Supplementary Table S1, S2).

We found that the high-income countries can supply adequate food nutrients to their population with domestic production in general, while food production in low-income countries are unlikely to safeguard population's nutrition requirements especially for calcium, choline, vitamin B12, PUFAs, and vitamin A (Table 2). From dietary nutrient perspective, Europe, North and South America tend to have adequate food production, except vitamin A for Latin America & Caribbean. In contrast, Middle East & North Africa and South Asia lack nutritious foods showing production-side gaps with respect to the population-level requirements of eight and 10 essential nutrients, respectively (Supplementary Table S3).

These results are in line with recent studies (19, 24, 25, 34) and illustrate the unequal distribution of food resources across the world and the need for nutrition-sensitive food production strategies in most countries. Our estimates on nutrient production adequacy at global level are smaller than those reported in a previous study (34). For example, the adequacy ratio for global protein production is 2.03 in this study (Table 1) but 2.86 in Geyik et al. (34). This is because unlike them, we additionally accounted for food loss, food waste and non-food use (seeds and other non-food products) in our net production estimates (Equation 1). Regarding the Population Share with Adequate Nutrient (PAN) indicator (Figure 3, Supplementary Table S4), our national values scoring domestic food production are lower or higher than those calculated with FAO Food Balance Sheet in Chaudhary et al. (28) depending on volumes of a country's imports and exports of food items.

Our analysis highlights the nutritional security concerns regarding the choline and PUFAs for which the current total production barely meets the global population requirements and most countries having inadequate national production amounts (Table 1). PUFAs are disproportionately underproduced in Middle East & North Africa (adequacy ratio = 0.38) and South Asia (0.41). This is important because previous global studies did not calculate the country-specific production amounts of PUFAs and choline (24, 34). An assessment based on the National Health and Nutrition Examination Survey (2009–2012 NHANES) found that the choline intake levels are well below the recommended levels in around 90% of Americans including most pregnant and lactating women (55), thus underscoring the need to increase awareness among health professionals and consumers regarding choline (22).

Our study come with several limitations and uncertainties that should be considered when interpreting the results. First, this study is based on the data on food production and domestic utilizations from FAO (36), and not all food items produced in each country have been reported there. For example, less utilized indigenous crops and foods from household gardens are missing in the global database and thus were not considered in our analysis, which may have resulted in a potential underestimation of the quantity of nutrients produced at the national level.

Second, our results on the adequacy ratio are limited in reflecting the food security status. An adequacy ratio equal to or above 1 should be interpreted with caution, because it has ignored the unequal food accessibility for individuals within a country due to the differences in income levels or other factors such as market distance. However, we report the indicator Population share with Adequate Nutrient (PAN) for each country and nutrient which considers the variance of inter-individual differences to inform the inadequacy risks (Figure 3). We could not employ the country- and nutrient-specific Coefficient of Variance (CV) for all nutritional factors in our PAN estimation due to the lack of data and this should be a future research front. Third, we acknowledge that dietary patterns with recent trends such as higher meat consumption and calorie intake may reflect the evolving human dietary needs that can establish other “requirement” context when analyzing the adequacy of foods (19, 56–58).

Despite the aforementioned limitations, our study presents novel information for food policy makers, accounting for the nutrient production quantity that can actually contribute to human nutrition. By linking this production quantity with the population needs, our assessment quantifies the magnitude of production-side nutrient gaps and surplus and identifies the specific nutrients in each country that need attention and whose domestic production should be increased through appropriate interventions.

Our findings highlight the need for agricultural and food systems to explicitly consider nutrients and not just focus on increasing yields or accounting for calorie sufficiency. Since most countries showed adequate production of energy and protein but not of several essential micronutrients, efforts should be made to increase the availability of key micronutrients such as calcium, vitamin A, and choline in most countries. The gaps associated with the inadequately produced nutrients can be narrowed through the yield gap closure (59), production of crops rich in these nutrients (60, 61), and biofortification (62, 63). Such interventions to improve nutrition security should also consider the linked impact on other sustainability elements (e.g., environmental impact, change of food prices, farmers' livelihood, etc.) to avoid trade-offs (9, 28).

It should be acknowledged that the international or interregional differences in the nutrient production capacity in many circumstances are constrained by environmental factors such as arable land, freshwater basins, heat, and precipitation, as well as social factors like labor costs and infrastructure (58). Food trade is thus crucial to help tackle the problems induced by the unequal distribution of agricultural resources and suitability as it can facilitate the transfer of foods with targeted nutrients from production surplus areas to areas with deficiency (24, 35).

Our results on the global hotspots of nutrient deficits or surpluses of the national food production systems (Table 1, Supplementary Table S3) together with nutritional decomposition at food group level (Supplementary Table S1) provide important information that can be used by businesses and governments for enabling targeted food trade (64), assessing the reliance on country's food imports (65, 66) and monitoring the risks from potential food supply shocks (67, 68). For example, a low-income country with low adequacy in essential nutrient production would be particularly vulnerable to domestic or external food supply changes due to their limited purchasing power in the international food market. A higher nutrition insecurity risk would be expected if the low-adequacy country relies on the food imports from countries having a sparse surplus in their food production.

The nutritional quality of diets for local and global consumers relies on the nutrient profiles of the outputs from the food production, albeit with varying and often considerable processing and transformation including trade (7, 35, 69). On the production side, more attention should be paid to the nutrients that are currently underproduced that could contribute better to nutrient intake adequacy at the national level. For example, production of foods rich in calcium, vitamin A, and choline in particular should be encouraged in low-income countries (Figure 1, Table 2). Population-adjusted nutrient requirements serve as the lower boundaries of the food system to provide diverse nutrients for health needs. A healthy and active life for everyone would be jeopardized if the system failed to meet these boundaries.

Future studies could adopt the framework demonstrated above to assess the food production and requirements in different contexts such as different population structures, alternative production systems, and food policies and to capture the nutritional performance of the crops, livestock, aquaculture and fishery systems. Combined with more complex modeling and data, our indicator can be applied to evaluate the projected or optimized food production with more efficient resource use, policy interventions and various sustainable transition scenarios or pathways.

Overall, our study contributes to the existing scientific calls for the transition from calorie-oriented food systems to a nutrition-sensitive system for achieving multiple Sustainable Development Goals (e.g., SDG 2 Zero Hunger and SDG 3 Good Health and Well-being) (1, 8, 61, 70).

## Data Availability Statement

The original contributions presented in the study are included in the article/Supplementary Material, further inquiries can be directed to the corresponding author.

## Author Contributions

AC: conceptualization, methodology, validation, formal analysis, investigation, resources, writing—original draft, writing—review and editing, supervision, project administration, and funding acquisition. CC: conceptualization, methodology, software, validation, formal analysis, investigation, resources, data curation, writing—original draft, writing—review and editing, visualization, supervision, project administration, and funding acquisition. AM: writing—review and editing, supervision, project administration, and funding acquisition. All authors contributed to the article and approved the submitted version.

## Funding

This research was funded by the National Research Program Sustainable Economy: resource-friendly, future-oriented, innovative (NRP 73) by the Swiss National Science Foundation (Grant number: 407340_172415) and the Initiation Grant of IIT Kanpur, India (project number 2018386).

## Conflict of Interest

The authors declare that the research was conducted in the absence of any commercial or financial relationships that could be construed as a potential conflict of interest.

## Publisher's Note

All claims expressed in this article are solely those of the authors and do not necessarily represent those of their affiliated organizations, or those of the publisher, the editors and the reviewers. Any product that may be evaluated in this article, or claim that may be made by its manufacturer, is not guaranteed or endorsed by the publisher.
